# Magnetization-polarization cross-control near room temperature in hexaferrite single crystals

**DOI:** 10.1038/s41467-019-09205-x

**Published:** 2019-03-18

**Authors:** V. Kocsis, T. Nakajima, M. Matsuda, A. Kikkawa, Y. Kaneko, J. Takashima, K. Kakurai, T. Arima, F. Kagawa, Y. Tokunaga, Y. Tokura, Y. Taguchi

**Affiliations:** 1 RIKEN Center for Emergent Matter Science (CEMS), Wako, Saitama 351-0198 Japan; 2 Neutron Scattering Division, Oak Ridge National Laboratory, Oak Ridge, TN 37831 USA; 3 Engineering R & D Group, NGK Spark Plug Co., Ltd., Minato-ku, Tokyo 108-8601 Japan; 4 Neutron Science and Technology Center, Comprehensive Research Organization for Science and Society (CROSS), Tokai, Ibaraki 319-1106 Japan; 5 Department of Advanced Materials Science, University of Tokyo, Kashiwa, 277-8561 Japan; 6 Department of Applied Physics, University of Tokyo, Hongo, Tokyo 113-8656 Japan

## Abstract

Mutual control of the electricity and magnetism in terms of magnetic (*H*) and electric (*E*) fields, the magnetoelectric (ME) effect, offers versatile low power consumption alternatives to current data storage, logic gate, and spintronic devices. Despite its importance, *E*-field control over magnetization (*M*) with significant magnitude was observed only at low temperatures. Here we have successfully stabilized a simultaneously ferrimagnetic and ferroelectric phase in a Y-type hexaferrite single crystal up to 450 K, and demonstrated the reversal of large non-volatile *M* by *E* field close to room temperature. Manipulation of the magnetic domains by *E* field is directly visualized at room temperature by using magnetic force microscopy. The present achievement provides an important step towards the application of ME multiferroics.

## Abstract (web summary)

Mutual control of the electric polarization and magnetization promises for low power consumption spintronic devices but remains challenging. Here the authors show reversal of non-volatile magnetization by electric field as well as the polarization switching by magnetic field in a single-component material, close to room temperature.

## Introduction

Multiferroic materials, endowed with both orders of polarization (*P*) and *M*, exhibit various intriguing phenomena due to the interplay of magnetic and electric degrees of freedom, such as *H*-induced *P* flop^1,2^, *E*-control of magnetic helicity^3,4^, and optical nonreciprocal directional dichroism^5,6^. The cross-coupling phenomena can greatly expand the functions of materials, and hence the multiferroic materials are anticipated to be applied to technological devices. In particular, nonvolatile, *E*-driven reversal of *M* without significant dissipation will lead to magnetic memory devices with ultra-low power-consumption^7–13^.

To accomplish this goal, strong coupling between *P* and *M* is necessary. In general, depending on the microscopic mechanism of *P* generation, the strength of the cross-coupling is different: In type-I multiferroics where *P* emerges potentially at a high temperature, but independently of the magnetic ordering, the coupling between *P* and *M* is weak, while in type-II multiferroics where *P* is induced by magnetic ordering, the coupling between *P* and *M* is strong^14^. Thus far, the *E*-induced *M* reversal has been investigated for both type-I and type-II mutiferroics. Heterostructures based on BiFeO_3_ belonging to the type-I category have been demonstrated to be promising^12,13,15,16^, while *H*-induced *P* reversal is difficult^17^. In the type-II category, good performance has been reported for hexaferrite materials^10,11,18–22^ with various structural types, including those at room temperature^23–25^. However, in previous studies^23,24^, ME effects with symmetric *P*−*H* and *M*−*E* field dependence were observed at room temperature, which unfortunately cannot achieve *E*-field-controlled magnetic memory function, or in another study^25^ the spontaneous *P* was found to diminish slightly below room temperature without any *M*−*E* control. Among the various compounds, the largest *M* switching (~3 *μ*_B_ per f.u.) by *E* was obtained in Y-type hexaferrites^10,11^ at cryogenic temperatures, which is attributed to the simultaneous reversal of the ferroelectric and ferrimagnetic order parameters in a particular multiferroic phase, termed FE3 phase. Moreover, this FE3 phase was found to emerge as a metastable state even at room temperature^22,26^.

Here we demonstrate that by choosing appropriate chemical composition and performing high-pressure oxygen annealing, the FE3 phase can be partially stabilized up to above room temperature. This enables us to observe reversal of *M* with considerable magnitude by *E* field as well as the nearly full reversal of *P* by *H* field in a single-component material near room temperature. By using magnetic force microscopy (MFM) technique, magnetic domain switching by *E* field is visualized and the fundamental insight into the *P*−*M* clamping is provided, which is expected to promote further advances in the magnetization-polarization cross-control.

## Results

### Structural and magnetic properties

**Fig. 1:**
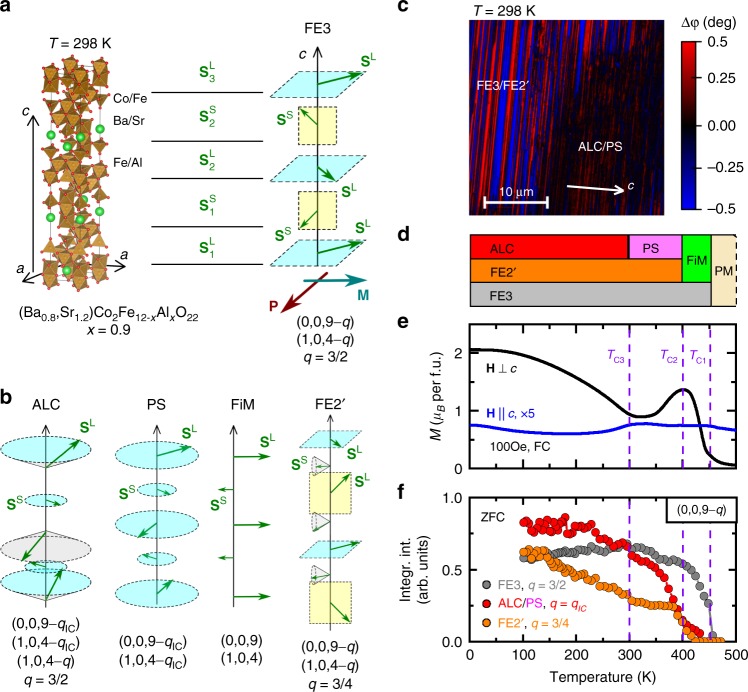
Structural and magnetic properties. **a**, **b** Schematic of the structural and magnetic unit cells of Ba_0.8_Sr_1.2_Co_2_Fe_12−*x*_Al_*x*_O_22_ (*x* = 0.9, BSCFAO), the latter of which is composed of alternately stacked spin-blocks with large (**S**^L^) and small (**S**^S^) magnetic moments. Alternate longitudinal conical (ALC), proper screw (PS), ferrimagnetic (FiM) orders as well as the multiferroic FE3 and FE2′ phases are illustrated in terms of the spin-blocks. The FE3 phase can be viewed as a double fan structure, where the **S**^L^ and **S**^S^ spins are staggered in the *ab* plane and the plane including *c*-axis, respectively, and the net magnetization (**M**) and net polarization (**P**) are perpendicular to each other and also to the *c-*axis. Respective magnetic phases are identified by the characteristic magnetic reflections indicated below each of the magnetic structure. **c** Real-space magnetic force microscopy (MFM) image of a BSCFAO sample with an *ac* surface after zero-field cooling. The striped and dark regions correspond to the magnetoelectric FE3/FE2′ and the incommensurate ALC/PS phases with typical dimensions of 5–30 μm with high and low contrast of MFM phase signals, respectively, indicating the phase separation. The magnetic domain within the FE3/FE2′ region has 200–300 nm in thickness along the *c-* axis and 10–20 μm in length along the *ab* plane. **d** Zero-field-cooled magnetic phase diagram presented as a chart diagram, showing coexistence of the magnetic phases. Horizontal axis is common for panels **d**–**f**. **e** Temperature dependence of the field-cooled *M* in *H* = 100 Oe for **H** || *c* and **H** ⊥ *c*. **f** The integrated intensities of selected neutron diffraction peaks representing the different magnetic phases in the zero-field-cooled measurements are plotted against temperature

Figure 1a shows the structural unit cell of the Y-type hexaferrite studied in the present work, Ba_0.8_Sr_1.2_Co_2_Fe_12−*x*_Al_*x*_O_22_ with *x* = 0.9 (BSCFAO), which is composed of Fe^3+^/Co^2+^ and Fe^3+^/Al^3+^ ions in tetrahedral and octahedral oxygen coordinations, respectively, similarly to the other members of the material family. It has been known^21^ that the magnetic structure in the hexaferrites is well described by ferrimagnetically ordered spin-blocks with large (${\mathbf{S}}_i^{\mathrm{L}}$) and small (${\mathbf{S}}_i^{\mathrm{S}}$) net magnetizations alternately stacked along the *c*-axis. As a result of complex magnetic interactions among the adjacent magnetic blocks, various magnetic structures have been identified^20,22^. These structures, such as commensurate phases FE3 and FE2′ (ref. ^20^), alternating longitudinal conical (ALC)^27^, proper screw (PS), and collinear ferrimagnetic (FiM) phases (schematically illustrated in Fig. 1a, b), are also observed in the present material. The magnetic ground state reached via zero-field cooling was reported to be ALC for a hexaferrite with a similar composition^28,29^. The FE3 phase is induced by *H* field applied within the magnetic easy-plane, but preserved as a metastable state even after the field is removed^22,26^. In the FE3 phase the magnetic moments of the **S**^L^ and **S**^S^ blocks form a double fan structure^22^, lying in the *ab* plane and a plane containing *c*-axis, respectively. Spin-driven *P* emerges within the *ab* plane and perpendicular to the net *M*, due to the inverse Dzyaloshinskii−Moriya mechanism^19,20^.

The magnetic phases in BSCFAO have been investigated by the zero-field-cooled (ZFC) MFM, low-field-cooled magnetization, and neutron diffraction measurements as shown in Fig. 1c–f. The neutron diffraction measurements revealed a complex magnetic phase diagram with several coexisting magnetic orders (Fig. 1d and see Supplementary Notes 3 and 4 and Supplementary Figs. 4 and 5), which was determined by taking also the previous results into account^20,22,30^. Below *T*_C1_ = 450 K, a magnetic peak with a commensurate wavevector of *q* = 3/2 appears together with the onset of *M* for **H** ⊥ *c*, which indicates the coexistence of the FE3 and the collinear FiM phases. At *T*_C2_ = 400 K, the magnetic peaks with commensurate *q* = 3/4 and incommensurate *q*_IC_ wavenumbers emerge, while *M* for **H** ⊥ *c* decreases, indicating that the FiM phase is turned into the PS and FE2′ phases. Finally at *T*_C3_ = 300 K, the PS order changes to the ALC phase as *M* for **H** || *c* shows a slight decrease. Real-space MFM image of an *ac* surface at room temperature indicates the phase separation between the strongly magnetic (large averaged-magnetization hosting) FE3/FE2′ and weakly magnetic (little averaged-magnetization hosting) ALC/PS phases as shown in Fig. 1c (for details see Supplementary Fig. 5). A prominent feature of the present-composition compound, being distinct from the previous report^22^ on a similar Y-type hexaferrite, is the presence of stable FE3 phase among the ZFC states.

**Fig. 2:**
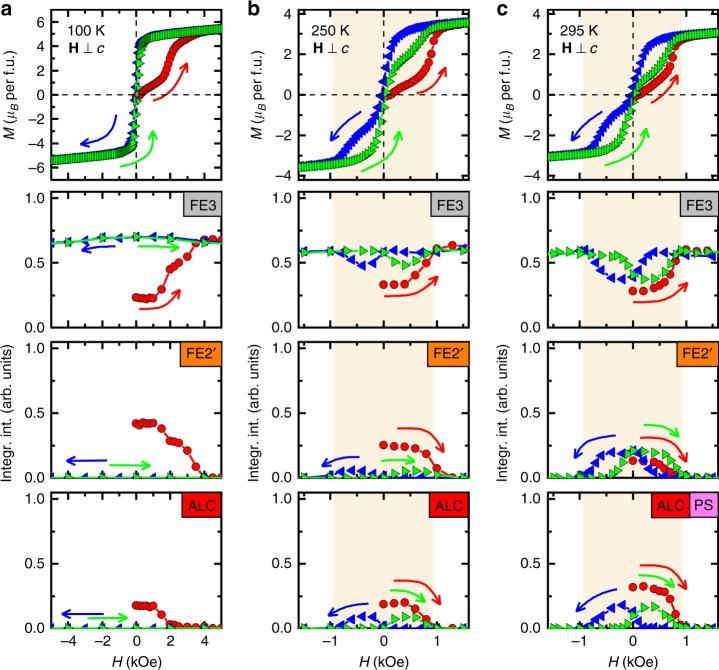
Magnetization and magnetic phases in magnetic field. Magnetic-field (*H*) dependence of magnetization and integrated intensity of neutron diffraction peaks for relevant phases at **a** 100 K, **b** 250 K and **c** 295 K. The measurements were started from a zero-field-cooled state, then *H* field was applied perpendicular to the *c-*axis. The FE3, FE2′, and ALC/PS phases are represented by the neutron diffraction peaks (0, 0, 9 − *q*) with *q* = 3/2, *q* = 3/4 commensurate, and *q*_IC_ incommensurate wavenumbers, respectively. In all the panels, red symbols indicate the initial *H*-increasing process, while blue and green symbols denote field-decreasing and second increasing runs, respectively. The ALC phase can be identified in the *M*−*H* measurements as exhibiting low *M* and low magnetic susceptibility (not shown) appearing at the low *H* region near the origin, while the FE3 phase has high *M*, showing distinction from the ALC phase. At *T* = 100 K (**a**), when magnetic field of 5 kOe is applied, the FE2′ and ALC phases disappear, leaving FE3 the only phase. Once the FE3 phase is stabilized, it is preserved throughout the subsequent reversal processes of the *H* field. At *T* = 250 K (**b**), the FE2′ and ALC phases re-emerge when the *H* field is reversed. The fraction of the re-appearing phases becomes largest not at zero field, but at ±500 Oe. Finally at *T* = 295 K (**c**), ratio of the re-appearing phases becomes larger, but the FE3 phase is partially preserved

Magnetic state under *H* applied within the *ab* plane was investigated by magnetization and neutron-diffraction experiments. In Fig. 2, *M* and the neutron diffraction intensities corresponding to each of the co-existing phases are separably plotted. Prior to the application of *H*, three phases coexist in the ZFC initial state in agreement with the temperature-dependent measurements. At 100 K (Fig. 2a), the ALC and FE2′ phases disappear at *H* = 2 kOe and *H* = 4 kOe, respectively, while the FE3 phase takes over their places. Once the single-phase state of FE3 is attained, it is fully preserved even when the *H* field is removed or reversed. On the contrary, at 250 K (Fig. 2b) and 295 K (Fig. 2c), both the ALC and FE2′ phases reappear upon the reversal of the *H* field. At relatively high temperatures, thermal agitation is large enough to overcome the energy barriers between the competing phases with almost degenerated free energies, while not at low temperatures. It is noted that magnetic anisotropy within the *ab* plane is negligible at room temperature (Supplementary Fig. 6), and hence the *M*−*H* curve as well as the diffraction intensity are least affected by the anisotropy.

### Magnetoelectric properties

**Fig. 3:**
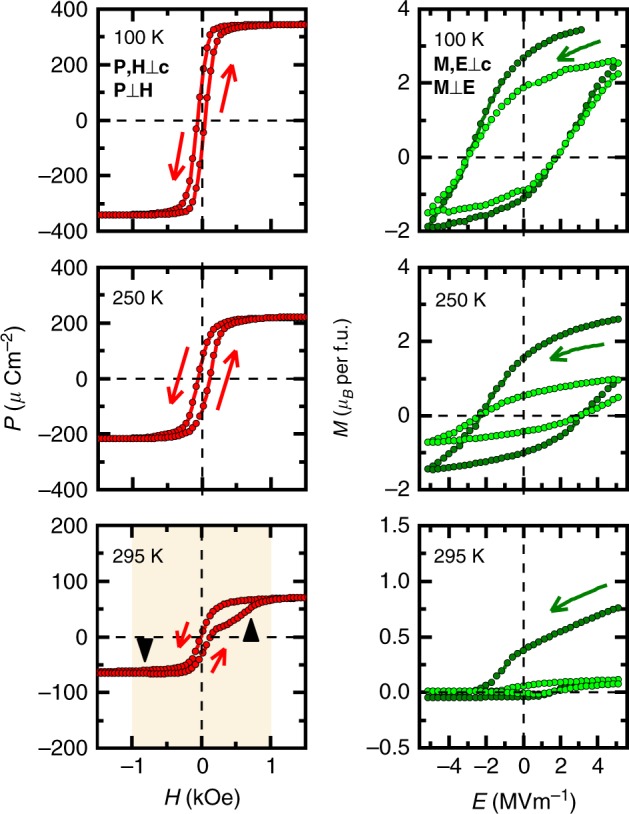
Cross-control of polarization and magnetization. Prior to the measurements, a single-domain ME state was prepared by (+*E*_0_, +*H*_0_) poling fields applied in **E** ⊥ **H**; **E**, **H** ⊥ *c*-axis configuration. The *P*−*H* and *M*−*E* measurements were performed in the absence of *E* and *H* fields, respectively. At low temperatures, both *P*−*H* and *M*−*E* curves exhibit antisymmetric shape with respect to the fields, indicating that the *P* × *M* is conserved due to the strong *P*−*M* coupling. Around room temperature, components with symmetric field-dependence are mixed in the *P*−*H* and *M*−*E* loops, implying that the *P* × *M* is not fully preserved anymore. The region where the ALC and PS phases re-emerge is highlighted by light shading in the *P*−*H* data at 295 K, while the secondary hysteresis of the *P*−*H* loops is indicated by black triangles

*H*-induced *P* and *E*-controlled *M* are shown in Fig. 3. Prior to the measurements, the single-domain ME state was attained by the application of (+*E*_0_, +*H*_0_) poling fields in a crossed configuration (**E** ⊥ **H**; **E**, **H** ⊥ *c*). Below *T* = 250 K both *P* and *M* show antisymmetric dependence on *H* and *E* fields, respectively, indicating that the *P*−*M* coupling is conserved throughout the reversal of the fields. Magnitude of the saturation value of the spin-driven polarization (*P*^sat^) is significantly larger than the earlier observations in other Y-type hexaferrites^10,19,22,31^, while comparable to TbMnO_3_^3^ and the spin-driven component of BiFeO_3_ which can be controlled by the field of more than *H* = 100 kOe ^17^. Correspondingly, the magnetization change between ±*E*_max_ fields, Δ*M*_E_ = 5.5 *μ*_B_ per f.u., at *T* = 100 K is larger than in any former experiments performed at lower temperatures^9–11^. Even at 250 K, a significant portion of *M* can be reversed (Δ*M*_E_ = 4.1 *μ*_B_ per f.u.) by the *E* field. Near room temperature, symmetry of the *P*−*H* and *M*−*E* loops begins to change to a symmetric butterfly shape, indicating that the *P*−*M* clamping is not fully preserved during the reversal. Moreover, *P*−*H* loops show a secondary hysteresis (indicated with black triangles), which is attributed to the re-emergence and disappearance of the PS and FE2′ phases as shown in Fig. 2c.

Importantly, the remanent *M* of BSCFAO can be switched in a nonvolatile manner between positive and negative values by *E* field even at 250 K, which is favorable for ME memory and spintronic applications. Changes in the remanent *M* for the first two *M*−*E* loops are as large as Δ*M*_1_ = 3.9 *μ*_B_ per f.u. and Δ*M*_2_ = 3.0 *μ*_B_ per f.u. at 100 K and Δ*M*_1_ = 2.5 *μ*_B_ per f.u. and Δ*M*_2_ = 1.5 *μ*_B_ per f.u. at 250 K. Correspondingly, the remanent *P* is also switched between positive and negative values with *P*^rem^ = 95 μCm^−2^ at 250 K. As for the retention, *P*−*H* loop exhibits good characteristics for the repeated reversal processes even at 295 K (see Supplementary Fig. 7). However, *M*−*E* loops are subject to deterioration at higher temperatures than 250 K. This decrease in the magnitude of the reversible *M* is attributed to the weakened *P*−*M* coupling as well as insufficient magnitude of the applicable *E* field.

### Investigation of the *P*−*M* coupling

**Fig. 4:**
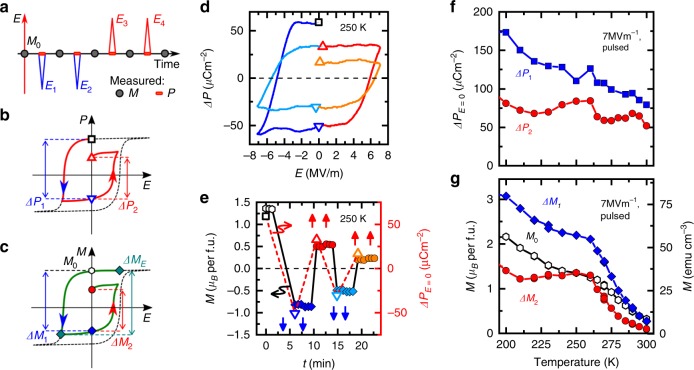
Magnetization reversal by pulsed electric field. **a** Schematic of the experimental procedure. Measurements were started from a single-domain ME state achieved by (+*E*_0_, +*H*_0_) poling in a [**E** ⊥ **H**; **E**, **H** ⊥ *c*] configuration. Triangular-shaped *E*-field pulses were applied to the sample. Magnetization was measured before and after the pulses, while polarization at the same time. Duration (*τ*) of the pulses was 50 ms and 1 ms for *T* < 280 K and $T\geqslant 280\,{\mathrm K}$, respectively. **b** Schematic illustration of the definitions for the Δ*P*_1_ and Δ*P*_2_ polarization changes. A minor loop without saturation of *P* is represented by red line. **c** Schematic *M*−*E* loop showing the definitions for *M*_0_, Δ*M*_1_, Δ*M*_2_ and Δ*M*_E_. A minor loop is illustrated by a green curve, *M*_0_ is the initial remanent magnetization after poling, and Δ*M*_1_, Δ*M*_2_ are the magnetization changes after −*E* and +*E* pulses. The Δ*M*_E_ is defined only for quasi-static experiments (Fig. S8) as the magnetization difference between +*E*_0_ and −*E*_0_ fields. **d** The polarization change of magnetic origin determined from the pulsed *E*-field experiments at 250 K after (+*E*_0_, +*H*_0_) poling. **e** Changes in the remanent *M* and *P* induced by the *E*-field pulses at 250 K. Blue downward and red upward arrows indicate the sign of the *E* pulses. Although both *M* and Δ*P*_*E* = 0_ decrease due to the insufficient *E*-field strength for complete reversal, their parallel change demonstrates their strong coupling. Temperature dependence of **f** Δ*P*_1_ and Δ*P*_2_, and **g** *M*_0_, Δ*M*_1_ and Δ*M*_2_ switched by the first and second *E*-field pulses. Up to 260 K, the *E*-field-induced Δ*P* and Δ*M* exhibit similar temperature variation. Upon approaching *T* = 295 K, temperature dependence of Δ*P* and Δ*M* shows clear departure, indicating that the *P*−*M* coupling starts to get weaker. From a technological viewpoint, the magnetization changes are shown also in emu cm^−3^ unit

To further clarify the behavior of the *P*−*M* coupling, *M* and *P* reversal was investigated simultaneously in pulsed *E*-field experiments (Fig. 4). Similarly to the quasi-static measurements, the single-domain FE3 state was initially prepared with (+*E*_0_, +*H*_0_) poling fields (**E** ⊥ **H**; **E**, **H** ⊥ *c*). After the removal of the poling fields, triangular-shaped *E*-field pulse pairs were applied antiparallel, then parallel with respect to the *E*_0_ poling field (see Fig. 4a). *M* was measured before and after the pulses, while *P* was measured during the same period as the *E*-field pulses were applied (see Methods). The Δ*P*−*E* curve of magnetic origin at *T* = 250 K is displayed in Fig. 4d, where the partial reversal of the ferroelectric *P* is attained by the pulsed *E* field.

Figure 4e shows simultaneous reversal of the remanent *P* and *M* by four pairs of *E*-field pulses at 250 K. Upon the first negative *E*-field pulse, both the remanent *P* and *M* change from positive to negative, causing the magnetization change Δ*M*_1_ = 2.3 *μ*_B_ per f.u. The *M* is almost completely reversed at this point, and there is only a small change in *M* for the second negative *E*-field pulse. For the subsequent two positive *E*-field pulses, *P* and *M* were again reversed from negative to positive, with the change of Δ*M*_2_ = 1.8 *μ*_B_ per f.u. Although the magnitudes of both Δ*P* and Δ*M* decrease as further pulses are applied, similarly to the quasi-static experiments, their parallel reduction demonstrates the strong *P*−*M* clamping in this temperature range (*T* ~ 250 K).

Temperature dependence of the *P* and *M* switched by the first negative and positive *E*-field pulses, as defined as Δ*P*_1_, Δ*P*_2_, Δ*M*_1_, and Δ*M*_2_ (Fig. 4b, c) respectively, are shown in Fig. 4f, g. Irrespective of the strength of the *P*−*M* coupling, *P* can be reversed by the *E* field. Magnitude of the reversed Δ*P*_1_ and Δ*P*_2_ slightly decreases as the temperature is increased, but remains finite at 300 K, since the multiferroic FE3 phase is present in the whole temperature region shown here. The initial value *M*_0_ and the switched magnetizations Δ*M*_1_ and Δ*M*_2_ show similar temperature dependence with the Δ*P* up to 260 K. In contrast to the Δ*P*, however, the Δ*M* exhibits more rapid decrease above 260 K, and almost vanishes at 300 K. Therefore, the *E*-control over the *M* is lost due to the weakened *P*−*M* coupling rather than to the reduced volume fraction of the FE3 phase.

### ME switching revealed by MFM at room temperature

**Fig. 5:**
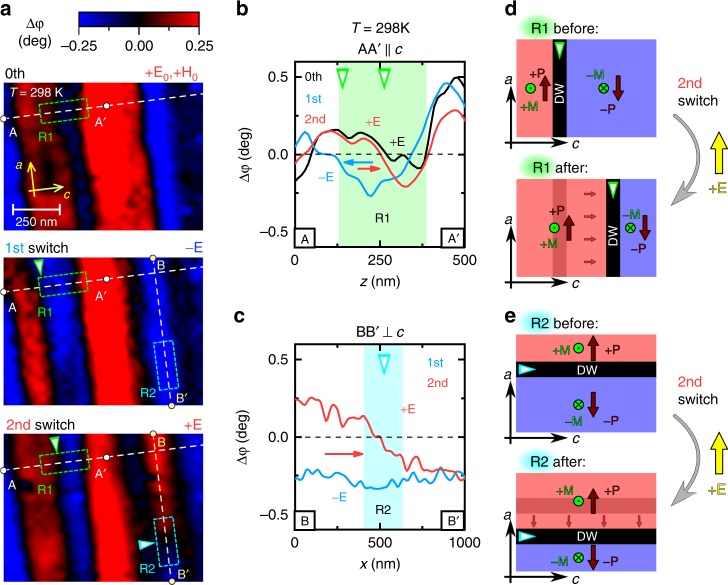
Real-space magnetic force microscopy (MFM) images. The MFM images were taken on the same 10 × 10 μm^2^ region of a BSCFAO crystal with an *ac* face (see Supplementary Figs. 3, 9 and 10) at room temperature. Prior to the MFM measurements, the sample was poled to a single-domain ME state using (+*E*_0_, +*H*_0_) poling fields in a **E** ⊥ **H**; **E**, **H** ⊥ *c* configuration. Panel **a** shows the changes in the magnetic domain pattern caused by two successive applications of the *E* field with different signs (the initial state is labeled as the 0th). The images include small regions, R1 and R2, where two representative cases of DW motion are observed. Around R1, the negatively magnetized domain (denoted with blue color, MFM phase shift Δ*φ* < 0) expands and shrinks along the *c*-axis upon the first and second applications of *E*-field, respectively. On the other hand, around R2, a positively magnetized domain (denoted with red color, Δ*φ* > 0) is pushed into the view area from the upper side along the *ab* plane. These two cases are further displayed as line profiles of the MFM phase shift (Δ*φ*) data along the **b** A−A′ and **c** B−B′ lines. Panels **d**, **e** show the schematic illustration of these two cases of domain wall motions for the second *E*-field switch, respectively

Using the real-space MFM imaging, we have investigated *E*-field-induced motion of the magnetic domain walls (DW) at room temperature (Fig. 5). The measurement was started from an initial state (0th in Fig. 5a), where poling *E* and *H* fields were once applied and then removed, and the evolution of the magnetic domain pattern of the same region was followed after several applications of the *E* field (1st–2nd in Fig. 5a and 1st–4th Supplementary Fig. 10). As displayed in Fig. 5a, magnetic domain pattern clearly shows changes in response to the applied *E* fields with different sign, which demonstrates that these are composite *P*−*M* domain walls^32^. The most typical cases of domain dynamics are observed in regions R1 and R2.

At region R1 in Fig. 5a, the negatively magnetized region expands and shrinks due to the successive applications of *E* field with alternate sign, which corresponds to DW propagation along the *c*-axis. Figure 5b shows the MFM signals taken along the A−A′ line, clearly demonstrating the DW motion along *c*-axis. The process of *M* switch is schematically illustrated in Fig. 5d, where one of the *M* domains expands along the *c*-axis, so that the ME domain with *P* parallel to *E* expands. The small magnetic anisotropy within the *ab* plane (see Supplementary Fig. 6) suggests that in this boundary between the oppositely magnetized regions, local net *M* is likely to rotate around the *c*-axis.

The region R2 in Fig. 5a, c exemplifies a different process, where a positively magnetized domain is pushed in the image area from the upper side. This behavior is clearly illustrated in the line profile of Fig. 5c. The process of *M* switch (shown in Fig. 5e) is similar to the previous case; however, in this case the domains are separated by a DW, where local *M* appears to form a cycloidal structure. Apart from these successful examples, change in the ratio between the majority and minority magnetic domains is relatively small, pointing to the decreased *P*−*M* coupling at a relatively high temperature, e.g. room temperature.

## Discussion

By choosing appropriate chemical composition and applying high-pressure oxygen annealing procedure, a multiferroic FE3 phase was stabilized up to 450 K in a single crystal of Y-type hexaferrite Ba_0.8_Sr_1.2_Co_2_Fe_12−*x*_Al_*x*_O_22_ with *x* = 0.9 (BSCFAO). At low temperatures, *P* and *M* are tightly clamped and reversed simultaneously, while at high temperatures, the *P*−*M* coupling becomes weaker, and the domain walls are deconfined although the multiferroic phase still survives. MFM measurements at room temperature demonstrated that the *M* switching by *E* field in BSCFAO is realized via the propagation of magnetic domain walls throughout the material. To further improve the *E*-field-induced *M* reversal in Y-type hexaferrites, the confinement−deconfinement crossover of the domain walls should be pushed to higher temperature, while the coexisting ALC, PS, and FE2′ phases have to be suppressed.

## Methods

### Single crystal growth and oxygen annealing procedures

Single crystals of Y-type hexaferrite, Ba_0.8_Sr_1.2_Co_2_Fe_12−*x*_Al_*x*_O_22_ with *x* = 0.9, were grown by the laser floating zone (LFZ) technique in 10 atm oxygen atmosphere. First, SrCO_3_, BaCO_3_, Co_3_O_4_, Fe_2_O_3_, and Al_2_O_3_ were mixed in stochiometric amount and sintered in air at 1150 °C for 24 h. Then the resulting product was pressed into rods and re-sintered for 14 h in the same conditions. Y-type hexaferrite single crystals from earlier growths were used as seeds for the LFZ growth. The single crystal rods were oriented with a back-scattering Laue camera and cut into discs with the surfaces containing *c*-axis. To increase the resistivity of the samples for the ME as well as neutron diffraction measurements, the cut pieces were annealed in 10 atm O_2_ at 1000 °C for 100 h in sealed quartz tubes, by adopting the technique described in ref. ^33^ (see Supplementary Notes 1 and 2 and Supplementary Figs 1–3).

### Neutron diffraction measurements

Neutron diffraction measurements were carried out at the triple-axis neutron spectrometer (PTAX) in the High Flux Isotope Reactor of Oak Ridge National Laboratory. Sliced and O_2_-annealed single crystal of BSCFAO (approximately 25 mm^3^) were placed in a cryomagnet with *H* applied along the [010] axis, while (*h*, 0, *l*) plane was set to be the scattering planes.

### *P*−*H*, *M*−*E* and *P*−*E* measurements

For each type of experiments, single crystals with the surface containing *c*-axis were coated with Au/Pt as electrodes; thus *E* field was applied in the *ab* plane, while *H* field was perpendicular to both the *E* field and the *c*-axis (**E** ⊥ **H**; **E**, **H** ⊥ *c*). *H* field dependence of the polarization was measured in a PPMS (Quantum Design) with an electrometer (Keithley 6517A) by monitoring the displacement current as the *H* field was swept with 100 Oe/s continuously between ±5 kOe for 11–21 cycles, depending on the signal to noise ratio. Current peaks around 0 Oe did not show degradation; therefore, the *P*−*H* curves were obtained by integrating the current after averaging. Magnetization measurement under *E* field was carried out in an MPMS-XL (Quantum Design), while the electrometer (Keithley 6517A) was used as a voltage source. The thickness, surface area, and mass of the sample were 70 μm, 1.64 mm^2^, and 0.71 mg, respectively. Pulsed *E*-field measurements were performed with a ferroelectric tester (Radiant Inc., Precision Premiere II) equipped with 500 V option. The ferroelectric polarization of magnetic origin was measured by the Positive-Up-Negative-Down (PUND) technique. At low temperatures, triangular-shaped *E*-field pulses with 7 MV/m in amplitude and 50 ms in duration were applied. Above 280 K, however, the pulse duration was reduced to 1 ms due to the lower resistivity (for further details see Supplementary Notes 5–8 and Supplementary Figs. 6–8).

### Magnetic force microscopy measurements

MFM measurements were carried out with a commercially available scanning probe microscope (MFP-3D, Asylum Research) using Co-coated cantilever (MFMR-10, Nano World). We used the two-pass technique (NAP mode in our MFM instrument of Asylum Research). Throughout the whole measurements, operation condition related to the cantilever was unchanged, which indicates no deterioration of it. For the analysis the linear flattening procedure was used. For *E*-field-dependent measurements, samples were poled to a single-domain ME state using +3 MV/m and +4 kOe poling fields in **E** ⊥ **H**; **E**, **H** ⊥ *c* configuration in a PPMS (for sample preparation see Supplementary Notes 5 and 9 and Figs. 5, 9 and 10). Static *E* field (+3 MV/m or −3 MV/m) was applied to manipulate magnetic domains using a Keithley 6517A electrometer, and the MFM images were taken after the *E* field was switched off. The sign and magnitude of the MFM phase shift, Δ*φ*, roughly correspond to those of the magnetization perpendicular to the plane.

## Supplementary information

Supplementary Information

## Data Availability

The data presented in the current study are available from the corresponding authors on reasonable request.
