# Supplementary material for: Magnetization-polarization cross-control near room temperature in hexaferrite single crystals
Source: Nat Commun. 2019 Mar 18;10:1247. doi: 10.1038/s41467-019-09205-x (PMC6423030; doi:10.1038/s41467-019-09205-x)
Supplement: Supplementary file 1 — Supplementary Information [file 41467_2019_9205_MOESM1_ESM.pdf]

Supplementary Information for "Magnetization-polarization cross-control near room temperature in hexaferrite single crystals"

V. Kocsis,[1] T. Nakajima,[1] M. Matsuda,[2] A. Kikkawa,[1] Y. Kaneko,[1] J. Takashima,[1,3] K. Kakurai,[1,4] T. Arima,[1,5] F. Kagawa,[1,6] Y. Tokunaga,[1,5] Y. Tokura,[1,6] and Y. Taguchi[1]

[1]*RIKEN Center for Emergent Matter Science (CEMS), Wako, Saitama 351-0198, Japan*
[2]*Neutron Scattering Division, Oak Ridge National Laboratory, Oak Ridge, Tennessee 37831, USA*
[3]*Engineering R & D Group, NGK SPARK PLUG CO., LTD., Minato-ku, Tokyo 108-8601, Japan*
[4]*Neutron Science and Technology Center, Comprehensive Research Organization for Science and Society (CROSS), Tokai, Ibaraki 319-1106, Japan*
[5]*Department of Advanced Materials Science, University of Tokyo, Kashiwa 277-8561, Japan*
[6]*Department of Applied Physics, University of Tokyo, Hongo, Tokyo 113-8656, Japan*

Supplementary Note 1: Effect of oxygen annealing on resistivity

Sliced samples were annealed in 10 atm O_2 at 1000 °C for 100 h in sealed quartz tubes, similarly to Ref. 1, by adopting the technique reported in Ref. 2 using Ag_2O as oxygen source. Figure S1 presents the electrical resistivity of BSCFAO measured by a two-probe method before (black curve) and after (red curve) the O_2 annealing procedure under constant voltage and 2 K min^{-1} temperature sweep rate. For the sake of comparison, the resistivity determined from the *I*-*V* curves simultaneously obtained during the *M*-*E* measurements at fixed temperatures are shown by orange coloured dots. Resistivity of BSCFAO shows semiconductor-like temperature dependence with an activation energy of 0.5 eV prior to the O_2 heat-treatment. After the the O_2 annealing, the activation energy is increased to 1 eV and the resistivity is enhanced by 5-6 orders of magnitude. The as-grown crystals have oxygen deficiencies, which indicates that the material has Fe^{2+} besides the Fe^{3+} ions, thereby leading to higher conductivity. The high-pressure annealing reduces the oxygen deficiencies and increases the resistivity.

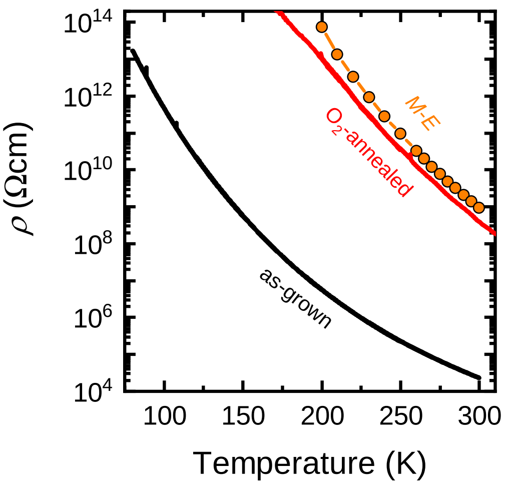

Supplementary Figure 1: | **Electrical resistivity of BSCFAO before and after O_2 annealing.** Resistivity measured in temperature sweep under constant voltage, before and after the high-pressure O_2 annealing, are presented by black and red curves. The orange symbols represent the resistivity obtained from the *I*-*V* curves at fixed temperatures during the measurement of the *M*-*E* loops.

Supplementary Note 2: Sample preparation procedures for magnetoelectric (ME) and magnetic force microscopy (MFM) mearurements

Sample preparation processes for the magnetoelectric measurements are illustrated in a step-by-step manner in Fig. S2a. Both for P-H and M-E measurements, single crystals were sliced so that the c-axis is parallel to the surfaces, which were coated with Au/Pt electrodes, thus E field was applied within the ab plane while the H field was perpendicular to both the E field and the c axis ($\mathbf{E} \perp \mathbf{H}$; $\mathbf{E}, \mathbf{H} \perp c$). In case of M-E measurements, coverage of the surfaces by the electrodes was crucial, as the M of the uncovered parts cannot be reversed. Therefore, the edges of the samples were cut off in order to ensure the complete coverage. The cut samples were placed on sapphire plates, where the bottom electrode was prepared with heat-treatment silver paste (Dupont 7095), while the top electrode was connected with a gold wire and silver paste (Dupont 4922N). A photograph of the sample used in the M-E measurements is shown in Fig. S2b. H field was applied perpendicular to the E field and the c axis along the lateral direction. This piece had 3 mm lateral dimension, 1.64 mm^2 surface area, 70 μm thickness, and 0.71 mg mass.

Photos of the sample for the E-field dependent MFM measurements are presented in Fig. S3. The sample was prepared with the following method. First, high-quality surface needed for the experiment was obtained by mechano-chemical polishing with 0.050 μm silica suspension on a BSCFAO sample with ac surface. According to the topology images, this polishing method provided surface roughness of ~3-4 nm. Grooves were cut parallel to the c axis in the surface with use of a wire saw (WS22, K.D. Unipress), and silver paste was used to form the electrodes and to fix gold wires. Distance between the electrodes in the present experiment is ~80 μm. During the poling procedure, the E=3 MVm^{-1} and H=4 kOe fields were applied within and perpendicular to the surface, respectively in the $\mathbf{E} \perp \mathbf{H}$; $\mathbf{E}, \mathbf{H} \perp c$ configuration, as shown in Fig. S3a.

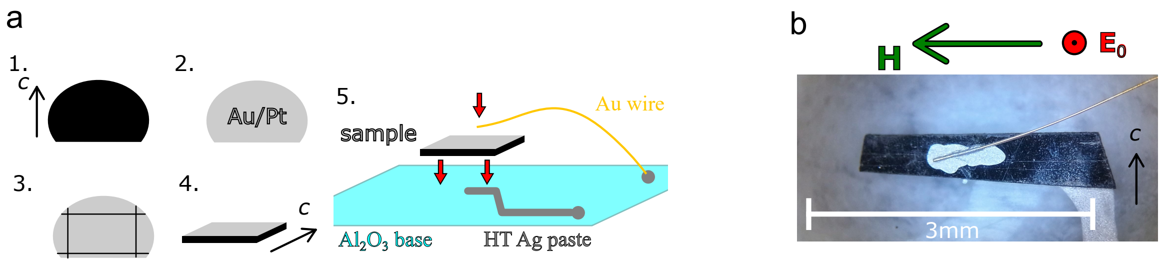

Supplementary Figure 2: | **Sample preparation procedure for the M-E measurements.** **a**, Schematic illustration of the sample preparation steps for M-E measurements. Polished plate with ~70 μm thickness (1) was covered with Au/Pt electrodes (2), then the edges were cut off (3-4). The cut pieces were fixed to sapphire plate with silver paste and Au wire (5). **b**, A photograph of the sample prepared for M-E measurements. Magnetic field was applied along the lateral direction of the sample, while E field was applied perpendicular to the plate.

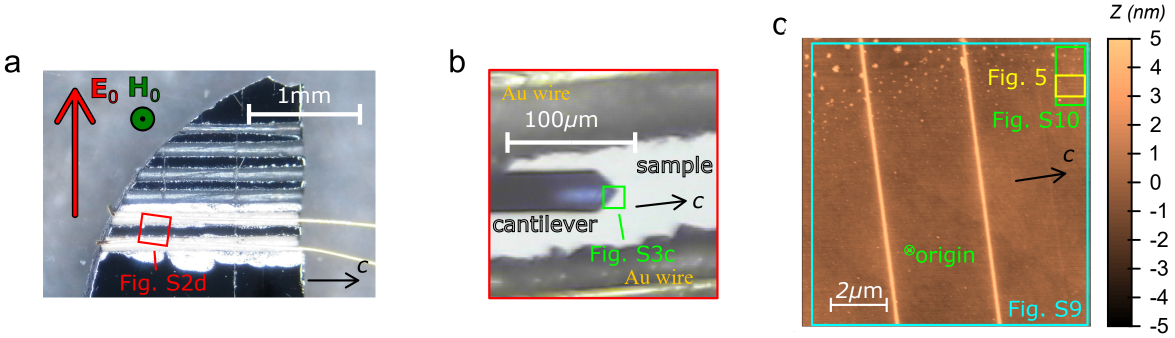

Supplementary Figure 3: | **The sample for the MFM measurements.** **a**, Optical microscope image of the BSCFAO sample with an *ac* surface prepared for the E-field dependent MFM experiments. E and H fields are applied within and perpendicular to the surface of the sample, respectively. Several grooves were cut so that they can be used to form electrodes. **b**, High magnification optical microscope image of the area of the measurement using the built-in camera of the MFM setup. The E-field dependent MFM images with $10 \times 10\,\mu m^2$ dimension were taken on the area labeled by a green square. **c**, Topography image, which is measured simultaneously with the MFM images. This area was chosen due to the fixed features (two parallel cracks and particles), which are easily recognized and makes it reproducible to re-locate the frame of the MFM measurement to the same area. MFM data were analyzed using the same feature (a small particle) common in each topography images as an origin.

Supplementary Note 3: Further details of the neutron diffraction measurements

Neutron diffraction measurements were performed on a thin plate (thickness $\sim$1 mm) of O_2-annealed single crystals with 25 mm^3 volume. Neutron diffraction profiles along $(0, 0, l)$ direction at selected temperatures and the corresponding wavenumbers of the FE3, FE2', ALC and PS magnetic orders are presented in Fig. S4a and S4b. In the paramagnetic phase, at T=500 K, only the two nuclear reflections are present at l=6 and l=9. At T=450 K, a strong magnetic peak appears at l=7.5, which identifies the FE3 phase with commensurate q=3/2 wavenumber ($l = 9 - q$). At around T=400 K, magnetic reflections with double-peak structure appear, corresponding to the commensurate q=3/4 and incommensurate q_{IC} wavenumbers. These magnetic reflections represent the FE2' and the ALC/PS phases, respectively. The q_{IC} wavenumber shows a weak and non-monotonous temperature dependence, while the wavenumbers of FE3 and the FE2' phases remain fixed to commensurate positions (Fig. S4b).

In Fig. S4e, temperature dependence of the neutron diffraction integrated intensities is displayed. The spin structures of the FE3 and FE2 phases are regarded as being composed of ferrimagnetic and cycloidal components that are parallel and perpendicular to the net magnetization direction, respectively, as depicted in Fig. 1 of Ref. 3. Therefore, the neutron intensities at $(0, 0, 9)$ and $(1, 0, 4)$ contain nuclear scattering and magnetic scattering from the ferrimagnetic component which has the same periodicity as the chemical lattice. As the temperature is decreased from 400 K to 300 K, the in-plane magnetization as well as the intensities at $(0, 0, 9)$ and $(1, 0, 4)$ are decreased, indicating that the q=0 ferrimagnetic components are reduced. On the other hand, the magnetic reflections corresponding to the wave vectors of $(0, 0, 3/2)$, $(0, 0, 3/4)$ and $(0, 0, q_{IC})$ remain unchanged or rather increase in this temperature range, suggesting that the reduction of the intensities at $(0, 0, 9)$ and $(1, 0, 4)$ are ascribed solely to the disappearance of the FiM order. The gradual increase below 300 K can be explained by an increase of volume fraction of the FE2 phase. In this series of Y-type hexaferrites, many previous studies have reported that PS or (alternating) longitudinal conical phase appears from the FiM phase as the temperature is lowered[4,5]. Although it could be possible to consider that the FiM phase still survives at low temperatures, the volume fraction of the residual FiM phase is likely to be very small. Therefore, the FiM phase can simply be viewed as a collinear parent phase, from which PS/ALC or FE2 emerges by spin canting or spin rotating at lower temperatures, and hence not relevant for the discussion on the observed magnetoelectric properties.

In Fig. S4f, half width at half maximum (HWHM) of each reflection is plotted against temperature. The $(0, 0, 9)$ nuclear peak serves as resolution limit for the HWHM of the magnetic peaks. As shown in Fig. S4f, the magnetic satellite peaks have larger HWHM than the nuclear peak, which implies the presence of magnetic domains. Moreover, respective magnetic satellite peaks have different HWHMs, indicating that the corresponding domain sizes may be different. If a third phase with multiple-q existed instead of phase separation, these correlation lengths should have been the same value. Therefore, phase coexistence of single-q magnetic phases is more likely.

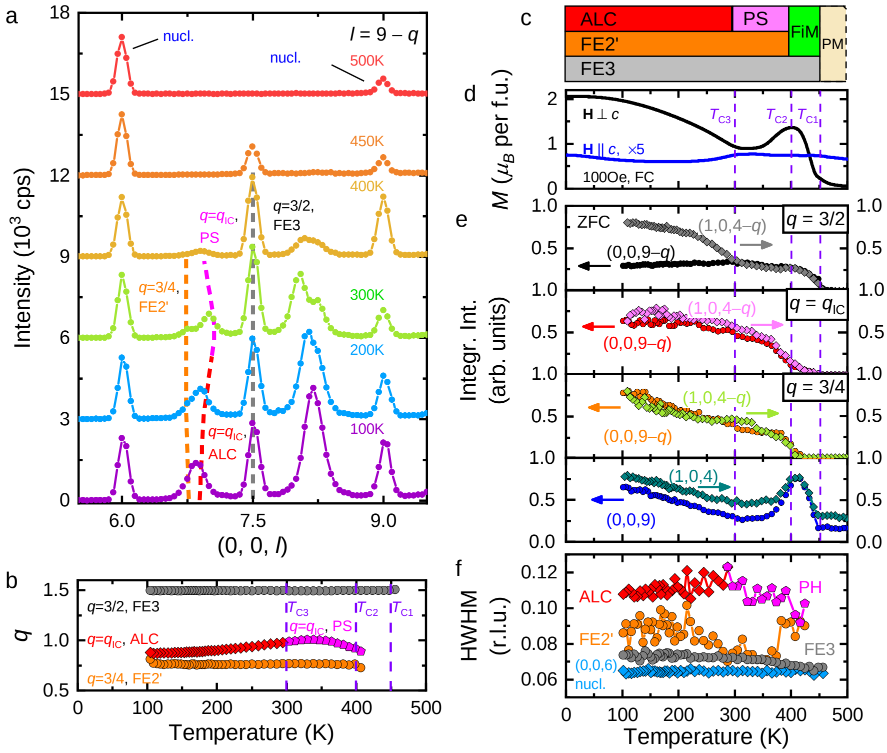

Supplementary Figure 4: | **Determination of the magnetic phases based on the neutron diffraction profiles.** **a**, Temperature dependence of the neutron diffraction profiles measured along the $(0, 0, l)$ line at H=0 Oe. **b**, Temperature dependence of the wavenumbers of the FE3, FE2', ALC and PS phases. Panel **c** is the magnetic phase diagram for zero-field-cooling and panel **d** shows the temperature dependence of the magnetization (the same as in Fig. 1 of the main text, re-plotted for better comparison). **e**, The integrated intensities of the neutron diffraction peaks plotted against the temperature. The integrated intensities are taken along the $(0, 0, l)$ and $(1, 0, l)$ lines in the zero-field-cooled runs. In the paramagnetic phase (T=500 K) only the two nuclear peaks at l=6 and l=9 can be observed. The FiM phase is signaled by the intensity change in the $(0, 0, 9)$ and $(1, 0, 4)$ reflections, while the FE3 phase, which is related to the magnetic peak with q=3/2 wavenumber ($l = 9 - q$), appears at T=450 K. The magnetic reflections with commensurate q=3/4 and incommensurate $q_{\rm IC}$ wavenumbers, corresponding to the FE2' and ALC/PS phases, appear around T=400 K. The FiM, FE2', and FE3 phases have ferrimagnetic component parallel to the net M, which contributes to the $(0, 0, 9)$ and $(1, 0, 4)$ peaks. The gradual increase below T=300 K can be explained by an increase of volume fraction of the FE2' phase. **f**, Temperature dependence of the half widths at half maximums (HWHM) of the $(0, 0, 6)$ nuclear peak and the magnetic peaks with q=3/2, q=3/4, and q=$q_{\rm IC}$ wavenumbers, corresponding to the FE3, FE2' and ALC/PS phases, respectively.

Supplementary Note 4: MFM measurements after zero-field-cooling

Zero-field MFM measurements were performed at room temperature on an O_2 annealed sample with mechano-chemically polished ac surface. This sample is different from the one used for the E-field dependent MFM experiments. General interpretation of MFM measurements is complicated, as even for an ideal (dipole) cantilever, it is not the surface magnetization but the $\frac{\partial^2 m_y}{\partial y^2}$ that is measured (y is the axis perpendicular to the surface). However in the present case, the observed striped domain pattern has similar dimension to the spatial resolution of the measurement, hence sign and magnitude of MFM phase shift, $\Delta\varphi$, roughly correspond to those of the magnetization perpendicular to the plane.

Figures 1c and S5 demonstrate the spatial separation of the coexisting magnetic phases in BSCFAO. The striped regions correspond to the phase with large M perpendicular to the surface of the sample (namely the FE3/FE2'), as the modulation of the MFM phase shift is large. On the other hand, the regions with low MFM signal are the incommensurate magnetic phases with small magnetization, namely the ALC/PS phases. Also, comparison between the MFM images of the ZFC (Fig. 1c) and poled (Fig. S9) states indicates that the dark contrast region is decreased after the poling with electric and magnetic fields. Assuming the above assignment of stripe domain region and dark contrast region to FE3/FE2 and ALC/PS phases, this observation is consistent with the neutron diffraction results as shown in Fig. 2c.

The magnetic phases were further investigated by changing the distance between the cantilever and the surface of the sample (Δh, representing the difference in the cantilever height between the topography imaging and the MFM

imaging). Figure S5b and S5c show the change in the MFM signal for different values of the Δh. By increasing the distance from the surface, magnitude of the MFM signal decreases, however, this change is larger at those regions where the modulation is originally higher (Figs. S5b and S5c). This suggests that in the areas with low modulation, an FE3/FE2' phase is buried beneath the the ALC/PS phase, as schematically illustrated in Figs. S5d and S5e.

In the present setup, the spatial resolution of the measurement is ~30 nm, while the observed stripes have 200-300 nm in width along the c axis and 10-20 μm in length along the ab plane. Therefore it is justified to assume that the striped pattern observed by the MFM measurement represents the magnetic domain pattern, while precise determination of the thickness along the c axis or the internal structures of the domain walls (DW) using MFM experiments alone is difficult.

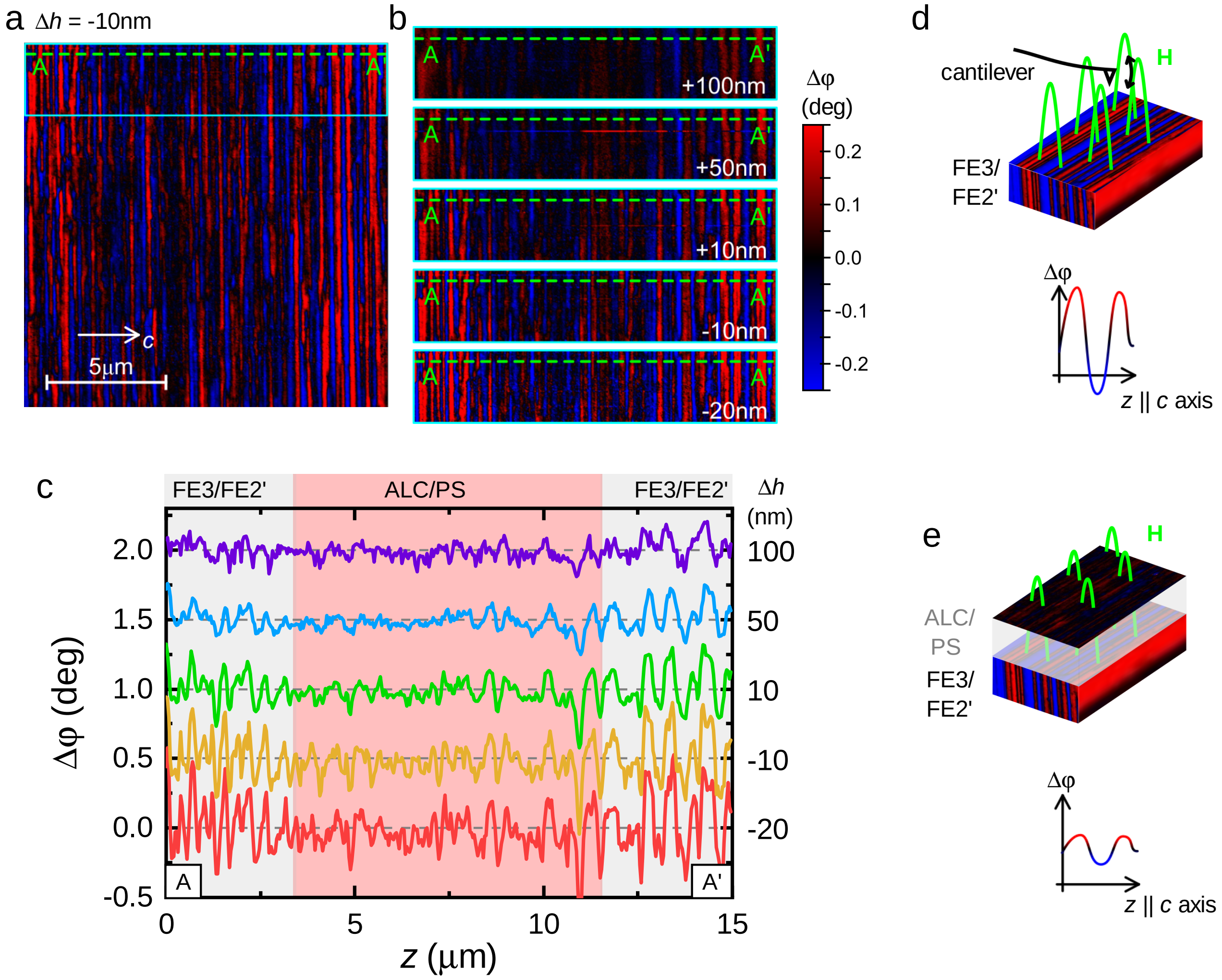

Supplementary Figure 5: | **The magnetic domain structures as investigated by MFM measurements.** **a**, An MFM image of a region with 15×15 μm^2 dimensions showing the FE3/FE2' (left and right sides) and ALC/PS phases (middle) with large and small modulation of the MFM phase shifts, respectively. **b**, MFM measurements for the same area (surrounded by light-blue rectangle in panel a) with different cantilever distance from the surface. Negative Δh means that the cantilever is closer to the sample surface during the MFM phase shift measurement than it was during the measurement of the topography. **c**, The MFM phase plotted along the line A-A' in panel a for various values of Δh. For each Δh, data are shifted by 0.5 deg for the purpose of clarity. Schematic figure of two MFM measurements, when the cantilever is above an FE3/FE2' phase at the surface (**d**), or above an FE3/FE2' phase covered with an ALC/PS phase at surface (**e**).

Supplementary Note 5: Magnetic anisotropy

Information on the magnetic anisotropy is useful for the understanding of the magnetic domain and domain wall structures. The FE3 phase of BSCFAO has easy-plane magnetic anisotropy perpendicular to the crystallographic c axis at T=300 K (see Fig. S6a). When the magnetic field is rotated within the easy-plane (ab-plane), a six-fold modulation is expected in the angular dependence of the M due to the trigonal crystal symmetry. As demonstrated in Figs. S6b and S6c, the magnetic anisotropy within the ab plane is negligibly small $\delta M(\vartheta) = M(\vartheta) - M_{\rm ave}$, where $M_{\rm ave}$ is the averaged magnitude of M over 360°. The six-fold modulation is as small as 0.1% of $M_{\rm ave}$ at 300 K and H=1 kOe, where the system is in the FE3 phase, and remains less than 0.7% at T=5 K. Therefore at room temperature, the net magnetic moment of the FE3 phase confined in the ab plane take arbitrary direction as a result of the small anisotropy within the ab plane.

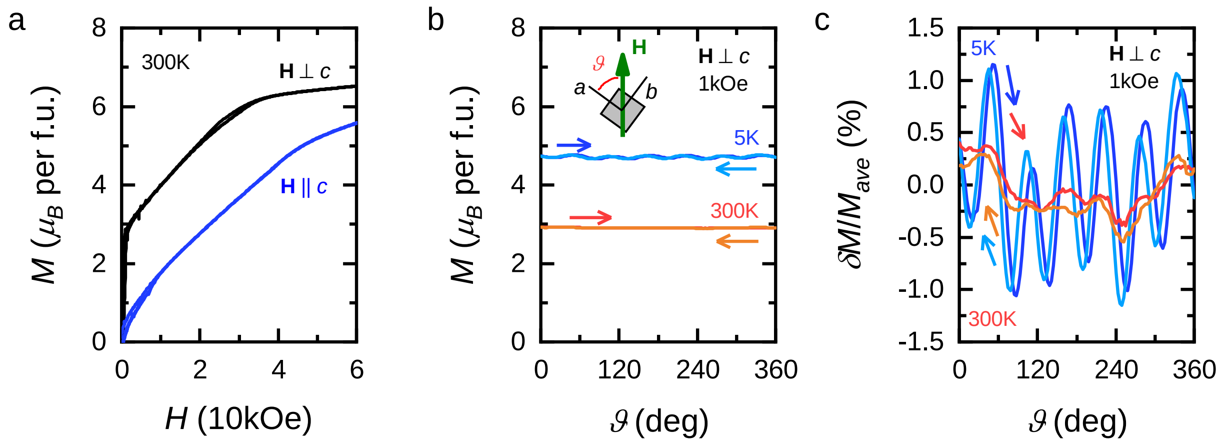

Supplementary Figure 6: | **Magnetic anirotropy of BSCFAO. a**, M-H curve of a BSCFAO sample at T =300 K demonstrating the strong easy-plane anisotropy. **b**, Field-orientation dependence of the magnetic moment at T =300 K and T =5 K. Moderate H field (1 kOe) is rotated within the ab plane, while the M is measured. **c**, Magnetic anisotropy, relative to the average of the M, was calculated from the data shown in panel b.

Supplementary Note 6: Magnetoelectric poling procedure

Prior to the P-H and M-E measurements, a single-domain FE3 state was prepared by isothermal ME poling with fields applied in the $\mathbf{E} \perp \mathbf{H}$; $\mathbf{E}, \mathbf{H} \perp c$ configuration. In this poling procedure, the single-domain FE3 state is attained by decreasing the H field, i.e. approaching from the high-field FiM phase, in the presence of E_0 field. In case of P-H measurements, $E_0 = 0.4\,\mathrm{MVm}^{-1}$ and $H_0 = 50$ kOe poling fields were applied at T=250 K, then the magnetic field was reduced to 5 kOe. After turning off the E field, temperature was swept to the measurement temperature in the presence of the 5 kOe field. In case of M-E measurements, the $E_0 = 5\,\mathrm{MVm}^{-1}$ and $H_0 = 10$ kOe poling fields were applied at the same temperature as the measurement was performed.

Supplementary Note 7: Measurement of H-dependent polarization

The spin-induced displacement current for repeated cycles at 295 K, used for the calculation of the P-H curve, is shown in Fig. S7. P-H measurements were started from a single domain ME state prepared by the isothermal poling procedure described before. The displacement current was monitored as the H field was swept between ±5 kOe with a rate of 100 Oe/s. When the H is reversed from positive to negative, a large negative current peak was observed, indicating the reversal of the P (Fig. S7a). For the $-H \to +H$ sweep, current pulse with double-peak structure was detected, which is associated with the reversal of P and the secondary hysteresis connected to the re-appearance of the FE3 phase. The displacement current was averaged for 21 cycles (orange curve) to obtain a single I-H loop (blue curve) in Fig. 7b, which was integrated to obtain P, and the resultant P-H curve is presented in Fig. 3b.

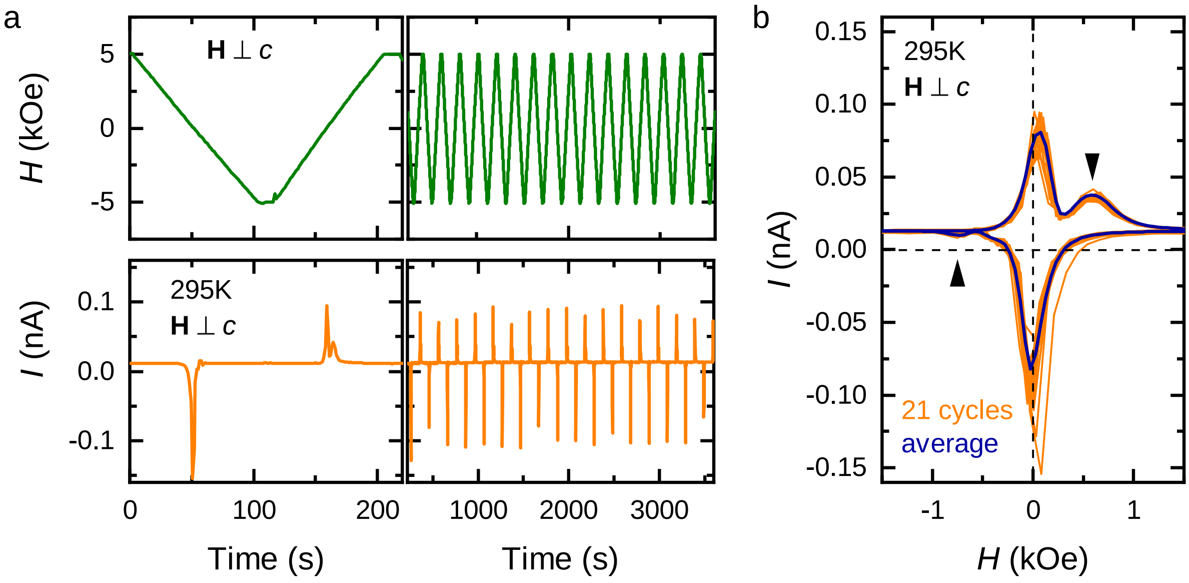

Supplementary Figure 7: | **Measurement of the spin-induced polarization at T=295 K. a**, Magnetic field dependence of the displacement current for the first cycle (left panel) and for subsequent cycles (right panel). Note the change in the scale of the horizontal axis. **b**, The measured displacement current (orange) was averaged for 21 cycles to a single I-H loop (blue). The averaged I was integrated to obtain P, and the resultant P-H curve is presented in Fig. 3b.

Supplementary Note 8: Temperature dependence of P-H and M-E properties in quasi-static measurement

Figure S8 shows the temperature dependence of the direct and the converse ME effects obtained from the quasi-static measurements. The saturation (P^{sat}) and remanent (P^{rem}) values of the spin-induced P, determined from P-H loops (see Fig. 3 and Fig. S7) are presented in Fig. S8a, while the initial M_0, the ΔM_1, ΔM_2 and ΔM_{E} changes, obtained from the M-E measurements (see Fig. 4c for the definitions), are shown in Fig. S8b. The P-H characteristics have different temperature dependence compared to the M-E characteristics, as discussed in the main text. ΔP_1 and ΔP_2 show gradual decrease towards 300 K, in contrast to P^{rem}, which completely disappears at 305 K. Magnitudes of the spin-induced P^{sat} and P^{rem}, as well as ΔP_1 and ΔP_2 are related to the volume fraction of the FE3 phase. However, P^{rem} and P^{sat} polarization values are those reversed by H-field, and hence should be affected by the P-M coupling, while ΔP_1 and ΔP_2 are not since they are driven directly by pulsed E-field, which explains the difference in the temperature dependence. Characteristic values associated with the M-E loops exhibit analogous temperature dependence between the quasi-static and pulsed E field measurements. Moreover, the initial M_0 and the change ΔM_{E} follow similar temperature dependence to the P^{rem} and P^{sat}, respectively.

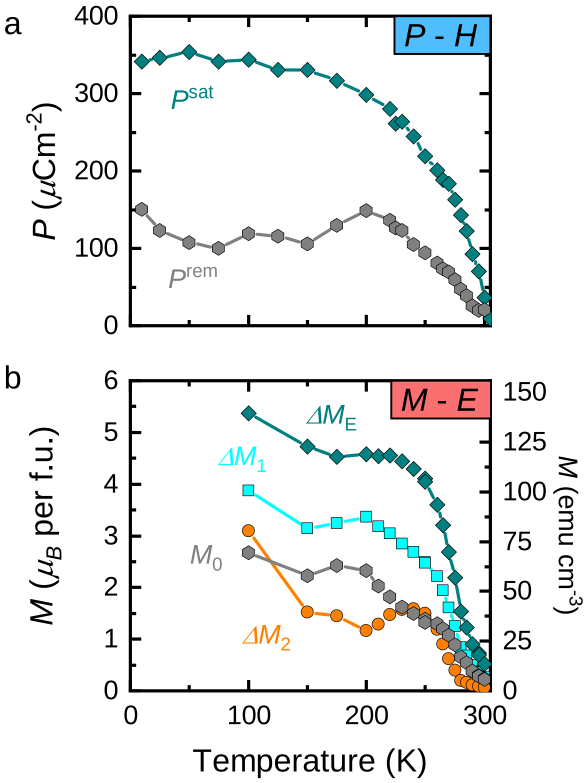

Supplementary Figure 8: | **Characteristic quantities in quasi-static P-H and M-E measurements as a function of the temperature.** **a**, The saturation ($P^{\rm sat}$) and remanent ($P^{\rm rem}$) values obtained from P-H measurement, have distinct temperature dependence from the ΔP_1 and ΔP_2 (see Fig. 4f) obtained from P-E loops. **b**, The M_0, ΔM_1 and ΔM_2 values measured in quasi-static E field sweep have similar temperature dependence to the corresponding values measured in pulsed E field (see Fig. 4). ΔM_E, the magnetization change between $\pm$5MV/m, follow similar temperature dependence to $P^{\rm sat}$. Due to its technological importance, M values are presented also in the unit of emu/cm^3.

Supplementary Note 9: E-field induced change in magnetic domains as revealed by MFM measurements

MFM measurements before and after the first application of E field of $-3\,$MVm^{-1} for the $10{\times}10\,\mu$m^2 area (shown in Fig. S3) are presented in Figs. S9a and S9b, respectively. The measurement was started from an ME-poled state with ($+E_0$,$+H_0$) fields in the $\mathbf{E}\perp\mathbf{H}$; $\mathbf{E},\mathbf{H}\perp c$ configuration. Magnitudes of the poling fields were E_0=3 MVm^{-1} and H_0=4 kOe. Figure S9c shows the changes between the switched and initial images as a difference between the MFM phases. The two MFM data were brought to complete overlapping using the same feature in their corresponding topography images as a point of reference (origin in Fig. S3c). Although Figs. S9a and S9b look rather similar at a first glance, Fig. S9c highlights that there are many differences all over the measurement area, demonstrating that magnetic domains respond to the applied E field, namely, there are many P-M clamped DWs. In particular, the MFM phase shift $\Delta\varphi$ integrated over a particular area shows good correspondence with the static M-E measurements.

Figure S10a shows the changes in the magnetic domain pattern for four consecutive applications of the E field in a particular area with $1{\times}2\,\mu$m^2 dimensions (see Fig. S3c). As described in the main text, the application of E field causes several changes in the magnetic domain pattern. Firstly, the DW parallel to the ab plane shifts along the c axis. The low anisotropy within the ab plane suggests that the net M is gradually twisted along the c axis within the DW. Secondly, a magnetization was reversed via the propagation of a DW within the ab plane. As the net M of the FE3 phase is strongly confined to the ab plane, the local M is twisted in a cycloidal manner within this type of DW. This type of DW is significantly wider ($\sim$500 nm, see Fig. 5c in the main text) than the DWs between the stripe domains along the c axis. Moreover, one example for reversible domain switching can be found in region R4 from the 2nd to the 4th switches in Fig. S10a. Both types of DW propagation contributes to the observed M reversal, and the former can change the thickness along the c axis of the domains, while the latter process can vary the area within the ab plane.

While there are many changes in the magnetic domain pattern, reversed magnetization is not so large at room temperature in the present compound. A possible explanation for this is provided by an example shown in Figs. S10b

and S10c. In region R3, the MFM phase takes zero value in a broad area after the first application of E field, and remains unchanged after the following switches. Figure S10d schematically illustrates what happens in this region. The DW between the FE3+ (positive magnetization) and FE3− (negative magnetization) domains is turned into the ALC/PS state. It is possible that the ALC/PS phase never returns to the FE3 phase in response to E field, therefore the M-E controllability is gradually deteriorated.

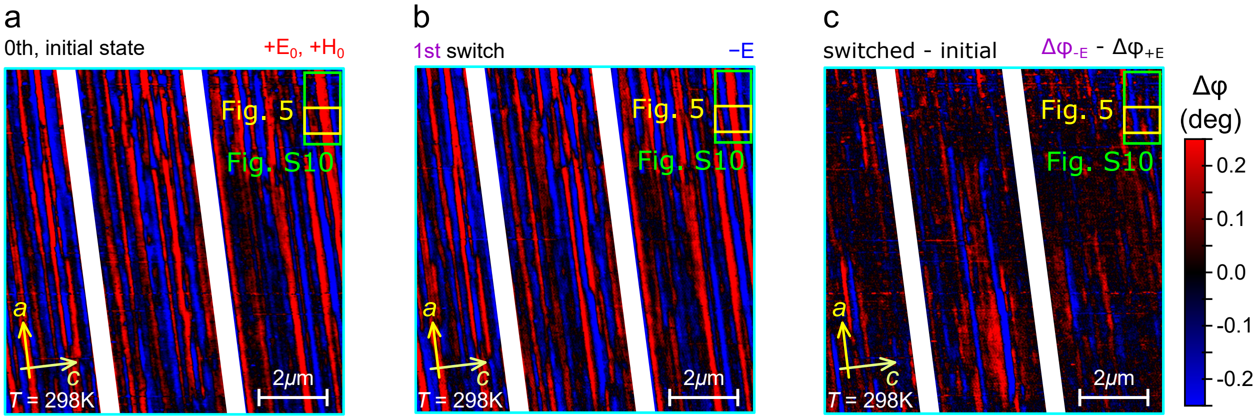

Supplementary Figure 9: | E **field manipulation of magnetic domains.** MFM images in a large area with $10\times10\,\mu\text{m}^2$ dimensions shown in Fig. S3c. The measurement was started from an ME-poled state, with the poling fields applied in the $\mathbf{E}\perp\mathbf{H}$; $\mathbf{E},\mathbf{H}\perp c$ configuration. During the measurements both E and H fields were removed and the E field switching was realized via the application of $-3\,\text{MV/m}$ static field. Panels **a** and **b** show the MFM phase shifts before and after the first application of E field of $-3\,\text{MV/m}$. The difference between the two images is shown in panel **c**. Slanted two blank areas in all the panels correspond to two scratches (see Fig. S3c), and hence MFM data are not available.

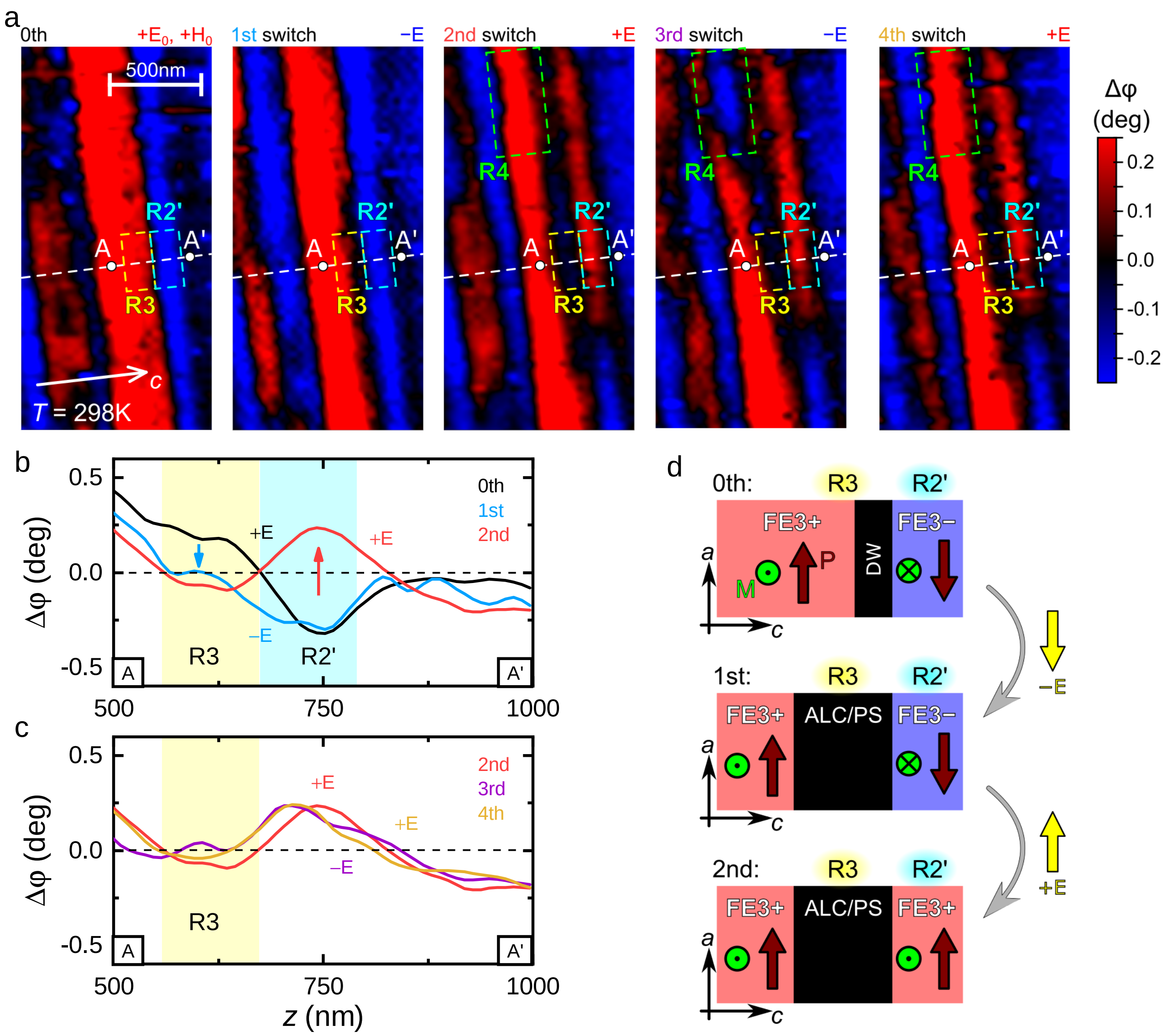

Supplementary Figure 10: | E **field manipulation of magnetic domains.** **a**, Evolution of the magnetic domain pattern for four consecutive applications of the E field. **b-c**, MFM phase along the line AA' before and after four applications of the E field. **d**, Schematic illustration of the MFM data at region R3. For the first applications of the E field, the DW between FE3+ (positive magnetization) and FE3− (negative magnetization) is turned into a weakly magnetic ALC/PS phase. For the second application of the E field, the FE3− domain is reversed to FE3+ at R2', which is caused by the propagation of DW along the ab-plane.

Supplementary References

---

[1] Hirose, S., Haruki, K., Ando, A. & Kimura, T. Effect of high-pressure oxygen annealing on electrical and magnetoelectric properties of $BaSrCo_2Fe_{11}AlO_{22}$ ceramics. *J. Am. Ceram. Soc.* **98**, 2104-2111 (2015).

[2] Inaguma, Y., et al. Synthesis and dielectric properties of a perovskite $Bi_{1/2}Ag_{1/2}TiO_3$. *Ferroelectrics* **264**, 127 (2001).

[3] Nakajima, T., et al. Magnetic structures and excitations in a multiferroic Y-type hexaferrite $BaSrCo_2Fe_{11}AlO_{22}$. *Phys. Rev. B* **94**, 195154 (2016).

[4] Lee, H. B., et al. Field-induced incommensurate-to-commensurate phase transition in the magnetoelectric hexaferrite $Ba_{0.5}Sr_{1.5}Zn_2(Fe_{1-x}Al_x)_{12}O_{22}$. *Phys. Rev. B* **83**, 144425 (2011).

[5] Lee, H. B., et al. Heliconical magnetic order and field-induced multiferroicity of the Co_2Y-type hexaferrite $Ba_{0.3}Sr_{1.7}Co_2Fe_{12}O_{22}$. *Phys. Rev. B* **86**, 094435 (2012).
